# A rare case of double pituitary prolactinomas: the diagnostic application of intraoperative ultrasonography and DNA methylation markers

**DOI:** 10.20945/2359-4292-2023-0506

**Published:** 2024-11-06

**Authors:** Jared C. Reese, Thomas M. Zervos, Jack Rock, Abeer Tabbarah, Houtan Noushmehr, Grayson Herrgott, Ana Valeria Castro

**Affiliations:** 1 Henry Ford Health Department of Neurosurgery Detroit MI USA Henry Ford Health, Department of Neurosurgery, Detroit, MI, USA; 2 Henry Ford Health Department of Pathology Detroit MI USA Henry Ford Health, Department of Pathology, Detroit, MI, USA; 3 Henry Ford Health Hermelin Brain Tumor Center Department of Neurosurgery Detroit MI USA Henry Ford Health, Omics Laboratory, Hermelin Brain Tumor Center, Department of Neurosurgery, Detroit, MI, USA; 4 Michigan State University College of Internal Medicine Department of Physiology East Lansing MI USA Michigan State University, College of Internal Medicine, Department of Physiology, East Lansing, MI, USA

## Abstract

The aim of this study is to describe the management and evolution of a patient with the rare condition of double lactotroph tumors and assess the role of intraoperative ultrasonography (IOUS) for their detection and methylation-based liquid biopsy for their diagnosis and monitoring. A 29-year-old woman diagnosed with double lactotroph tumors through hormonal and MRI workup underwent surgical resection due to cabergoline intolerance. To detect a tumor missing through visual inspection, IOUS was performed. Pituitary tumor (PT) and nontumor (NT) tissues and blood were collected for pathological and molecular assessments (genome-wide methylation level profiled using the EPIC array, at surgery and follow-up). Reference methylome data were obtained from publicly available repositories. Both tumors (T1 and T2) were detected via IOUS and confirmed as lactotroph tumors through immunohistochemistry. In tissue specimens, PT-specific markers distinguished T1 from NT tissue, while T2, primarily nontumor cells, clustered with NT specimens. In liquid biopsies, these markers differentiated between T and NT cohorts. During the 12-month follow-up, methylation profiling and prolactin blood assessments showed that methylation markers clustered with NT specimens, which coincided with prolactinemia normalization, indicating successful tumor control after surgery. This case illustrates the translational use of methylation-based liquid biopsy methodologies in detecting and monitoring PTs through the detection of tumor-specific markers in blood specimens. This approach can be useful to distinguish sellar masses mimicking PTs based on nonspecific imaging features and to monitor for early recurrence of PTs, particularly nonfunctioning PTs lacking specific biochemical markers. This case also illustrated the role of IOUS in identifying multiple PTs missed by visual inspection alone, leading to improved patient outcomes through complete tumor resection.

## INTRODUCTION

Isolated pituitary tumors (PTs) are the third most common intracranial neoplasms (approximately 15%) (1,2), with an approximate incidence of 4.0/100,000/year and a prevalence between 1:909-1:818 reported in population-based studies (1). Among PTs, multiple PTs – characterized by the co-occurrence of two or more separate and well-delineated pituitary lesions – exhibit an estimated prevalence of 0.2%-2.6% in surgical cases and 7.0%-10.5% among autopsied adenomatous pituitaries (3,4).

Multiple PTs encompass the combinations of various PT subtypes, stained for different hormones or cell-lineage-specific transcription factors (TFs). Most frequently, one of the components of multiple PTs is a growth hormone (GH)- or adrenocorticotropic hormone (ACTH)-producing tumor, while the other component includes tumors staining positive for prolactin, thyroid-stimulating hormone (TSH), follicle-stimulating hormone (FSH), and/or luteinizing hormone (LH), with confirmed multiple lactotroph tumors being rare among those (3-10). Multiple PTs typically result in hormonal excess caused by at least one of the tumor components (3-6). Achieving a biochemical cure or overcoming medical treatment resistance might require complete removal of all identified possible tumors, which is particularly crucial for patients with multiple PTs causing Cushing's disease or acromegaly (3-6). Preoperative and intraoperative recognition of double tumors through magnetic resonance imaging (MRI) can be challenging due to this method's limited sensitivity, and intraoperative visual inspection can fail to identify small tumors potentially impacting the treatment outcomes of these patients (3,11-15). In some cases, multiple PTs are only identified during pathological assessment (3). Although literature reviews indicate that intraoperative ultrasonography (IOUS) for pituitary surgery can be helpful in identifying anatomical structures in the sellar region and assessing the extent of resection, the Congress of Neurological Surgeons did not find sufficient evidence to recommend its use as part of pituitary surgery care. However, IOUS could be beneficial in selected cases, such as for patients with Cushing's disease whose microadenoma is not detected through intraoperative visual inspection (3,11-15).

Whether synchronous multiple PTs originate from different cell lineages (5,6) or from the transformation of one tumor cell lineage along a divergent path is still disputable (4). Accurately classifying the subtypes of tumors composing multiple PTs is crucial for precise diagnosis and effective treatment strategies. As methylation patterns reflect the cell of origin, the application of molecular methodology, such as DNA methylation profiling, becomes invaluable in this context both for diagnostic and surveillance purposes (16-23). This becomes especially critical in patients initially diagnosed with prolactinoma or somatotroph adenoma, whose growth does not respond to current drug treatments as anticipated, potentially leading to a misdiagnosis of treatment resistance (24,25). In such cases, the unresponsive tumor may not necessarily indicate therapeutic resistance, but the presence of a different subtype (*e.g.*, silent corticotroph tumor). These alternative subtypes may remain dormant or exhibit more aggressive growth in the future, underscoring the necessity for active surveillance, accurate identification, and tailored management (26,27).

In the present study, we aimed to achieve the following objectives: 1) describe the rare finding of synchronous double lactotroph tumors, 2) highlight the critical role of IOUS in detecting multiple tumors, and 3) describe the pituitary-specific methylation signatures detected in liquid biopsy for diagnosis and follow-up.

## CASE PRESENTATION

**Figure 1:**
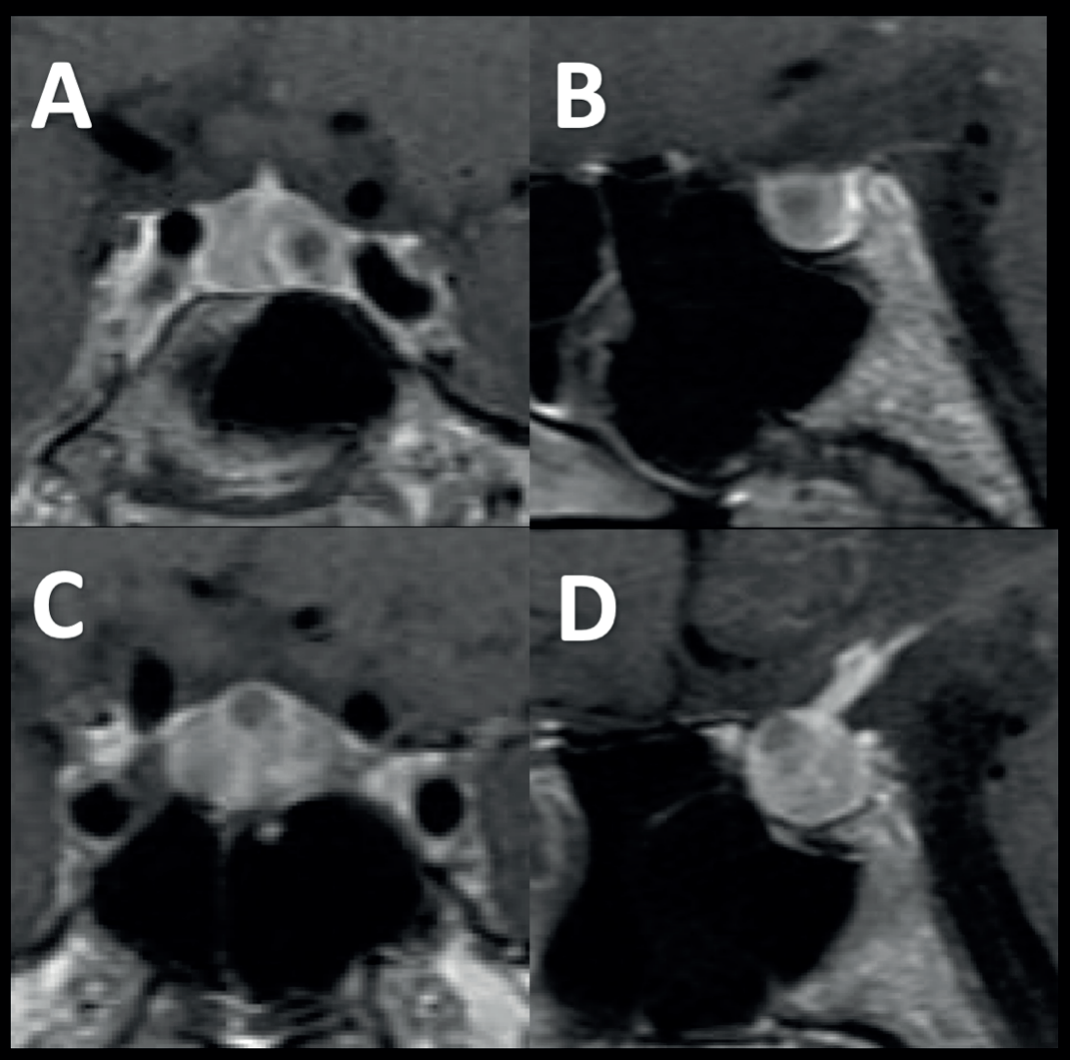
Magnetic resonance imaging (MRI) scan. Coronal and sagittal T-1 weighted MRIs showing an inferolateral pituitary tumor (images **A** and **B**) separated from a superomedial pituitary tumor (images **C** and **D**).

A 29-year-old woman with an initial history of menstrual irregularity and galactorrhea and high prolactinemia levels (not reported) was diagnosed with a lactotroph tumor and treated with cabergoline, which was suspended due to the development of mood disorder with its use. Due to this side effect and desire of the patient, she was referred to our service for surgical treatment. The patient did not report the administration of any other medications. Furthermore, there was no indication of a personal or familial history suggestive of tumors associated with multiple endocrine neoplasia type 1 (kidney stones, diarrhea, or hypoglycemia). Physical examination showed full visual fields and no detectable neurological deficit. An MRI revealed two separate Knosp grade 1 pituitary microadenomas (Figure 1). Except for blood prolactin assessed after cabergoline treatment (53.2 ng/mL, normal range [NR] < 27.0 ng/mL), all other hormonal tests were within normal limits (Table 1).

**Table 1:** Hormonal and biochemical workup before and after surgery

Features	Prolactin(ng/mL	TSH(mIU/mL)	Free T4(ng/dL)	IGF-1(ng/mL)	LH(mIU/mL)	FSH(mIU/mL)	ACTH(pg/mL)	Cortisol(µg/dL)	Ionized calcium(mmol/L)
Pre-surgery (cabergoline)	53.2	1.53	1.07	191	7.7	5.6	23	13.3	1.07
Post-surgery (12 months)	12.6*	1.46	1.32	122	8.1	6.4	28	13.8	–
Normal reference	<27.0	0.45-5.33	0.61-1.44	84-281	2.1-10.9	3.8-8.8	7-63	4.3-22.4	1.00-1.35

* 6 months: 6.3 ng/mL

The patient was treated via an endoscopic endonasal transsphenoidal approach for resection of the tumors. Intraoperative visual inspection failed to identify one of the two tumors previously shown in the preoperative MRI and IOUS was performed and corroborated the MRI findings by showing two distinct lesions separated by apparently normal pituitary tissue. Four solid tissue biopsies were performed and sent for immunohistochemistry and epigenomic analyses. Blood was also obtained preoperatively and 12 months postoperatively for hormonal and epigenomic profiling. After 16 months of follow-up without any additional treatment, the patient continued presenting normal pituitary function and prolactinemia levels, without indication for follow-up MRI.

## METHODS

The molecular profiling conducted on both tissue and liquid biopsy specimens followed the protocol approved by the Institutional Review Board of the Henry Ford Health Hospital (HFHS IRB# 10963; 12490; 12684). The patient provided informed consent for the utilization of tumor and liquid biopsy specimens for research purposes.

### Intraoperative ultrasonography

For the PT resection procedure, we performed a two-nostril approach used in our endoscopic surgeries and as detailed by others (13). The IOUS and the endoscope devices were separately introduced into each nostril at various times during the procedure. After exposing the pituitary gland through a transsphenoidal approach, a minimally invasive transducer designed for transsphenoidal and skull base surgery was employed (N20P6-9007, BK Medical, Denmark; frequency range 20-6 MHz, contact surface 6×7 mm, shaft length 150 mm, phased array). After removing the sellar bone, and prior to opening the dura, we obtained real-time imaging that allowed the visualization of surrounding structures (*e.g.*, optic nerves and blood vessels) and the tumors within the gland. These images guided the endoscopic neuronavigational system and facilitated the precise localization and confirmation of the complete removal of the tumors, including the smaller tumor that was not detected by visual inspection.

### Histopathological and immunohistochemical assessment

Pathological analysis was performed in four formalin-fixed, paraffin-embedded (FFPE) samples: two from the tumors (Tumors #1 and #2) and two from the apparent nontumor adenohypophysis in between the tumors (Nontumors #1 and #2). The FFPE specimens were sliced into 2-μm thick sections and stained with hematoxylin and eosin (H&E). The tissue was also subjected to immunohistochemical analysis exclusively for adenohypophyseal hormones (transcription factor assessment unavailable): GH, prolactin, TSH, ACTH, FSH, LH, alpha-subunit, reticulin, synaptophysin, and the proliferation marker Ki67.

### Tissue and liquid biopsy DNA methylation profiling

Four paraffin blocks containing distinct tissue segments of the two PTs and the two nontumor pituitary samples were suitable for DNA methylation profiling. For DNA extraction from the FFPE tissue, 10-µm curl sections were sliced. Genomic DNA was extracted from FFPE specimens using the QIAamp DNA FFPE Tissue Kit (Qiagen), according to the manufacturer's instructions.

Peripheral blood (15 mL) was drawn at the time of the surgical procedure (before the tumor excision) and at 12 months after surgery. Plasma and serum were separated within 1 hour from blood collection. Cell-free (cf)DNA was extracted from serum/plasma as described in our previous publications (16,17,22). Briefly, DNA from all serum/plasma samples was extracted from 1 mL (20 ng) of serum/plasma using the Quick-cfDNA Serum & Plasma Kit according to the manufacturer's protocol (Zymo Research - catalog # D4076). The DNA concentration was measured with Qubit spectrophotometer (Thermo Fisher Scientific) or 4200 TapeStation (Agilent Technologies) and was calculated by dividing the total amount of cfDNA extracted by the amount of serum/plasma used for extraction. Prior to profiling, the isolated and fragmented DNA was restored and concentrated as previously described (16,17,22).

The DNA extracted from FFPE and liquid biopsy specimens were bisulfite-converted (EZ DNA methylation Kit; Zymo Research) and profiled using an Illumina Human EPIC array (HM850K) at the USC Epigenome Center, Keck School of Medicine, University of Southern California, Los Angeles, California.

### DNA methylation analyses

The quality of DNA methylation data of FFPE specimens was determined using the BioTek Cytation 3 Imaging Reader (BioTek, Vermont, USA), and the quality of liquid biopsy specimens was assessed using shinyMethyl, a bioconductor package for interactive quality control of DNA methylation data from Illumina arrays, as previously described (16,17,22,23).

The arrays’ data from FFPE and liquid biopsy specimens were processed and analyzed using R (version 4.3.0) and the minfi (version 1.46.0) packages, as we previously detailed (16,17,22,23). Before analysis, we removed probes designed for sequences with known polymorphisms (SNPs), probes with poor mapping quality, probes with missing values, and probes located within either the X or Y chromosome, as we previously reported (23).

For tissue and liquid biopsy molecular reference, we downloaded tissue and liquid biopsy methylome data from a publicly available data repository (Gene Expression Omnibus [GEO]) containing data from a multicenter cohort representative of all PT subtypes and nontumor pituitary specimens (17,23). We aligned our FFPE methylomes profiled with the Illumina 850K with the publicly available tissue methylome profiled with Illumina HM450K (17,23) and identified and analyzed 393K CpG probes shared across both methylation platforms.

### Identification of pituitary tumor-specific methylation signatures

In order to identify PT-specific methylation signatures, we conducted a supervised analysis comparing the methylome of PTs and nontumor tissue cohorts (namely, tissue-reference cohort) generated in-house or extracted from public repositories (21,23). We selected signatures that exhibited differential CpG methylation levels between the groups through a two-sided Wilcoxon rank-sum test and selected significantly differential probes with false discovery rate (FDR)-adjusted p values < 0.001 and a differential methylation mean between > 0.3 and 0.375 (23). Our patient's FFPE-derived methylome was overlaid with the tissue-reference cohort for visualization purposes.

To scrutinize whether the identified tissue-derived PT-specific signatures were captured in liquid biopsy samples, we overlapped these probes with the blood methylomes across our liquid biopsy reference cohort methylomes derived from nontumor and PT samples (16,17,22). Then, these overlapping signatures were analyzed for differential methylation levels across our liquid biopsy reference through identical statistical methods used across the tissue, selecting signatures that displayed differential methylations across serum and plasma specimens. The patient's liquid biopsy methylomes (surgical and follow-up collections) were overlaid with the liquid biopsy reference cohort methylomes for visualization purposes.

### Data visualization

For visualization purposes, we used dimensionality reduction techniques specialized for high-dimensional and low-dimensional datasets – *e.g.*, principal component analysis (PCA) – across the reference methylome data and the patient's samples.

## RESULTS

### Pathology reports

Tumor #1 was composed of 90% adenoma tissue; it was densely granulated, stained positively for prolactin and synaptophysin and negatively for other adenohypophyseal hormones, and presented a proliferation index (MIB-1) < 1%. Tumor #2 was composed of 20% adenoma tissue (the remaining 80% comprised nontumor adenohypophyseal tissue); it stained positively for prolactin and negatively for reticulin and other adenohypophyseal hormones. Although MIB-1 assessment of Tumor #2 was attempted, it was deemed non-contributory due to tissue depletion. Nontumor #1 and #2 specimens were composed entirely of nontumor adenohypophysis displaying variable expressions of different hormones – mostly LH and FSH, followed by GH, TSH, ACTH and, less frequently, prolactin and alpha-subunit.

### Methylation markers

The FFPE and liquid biopsy samples presented sufficient and qualitative genomic (tissue) and cfDNA (liquid biopsy) material to profile genome-wide DNA methylation levels.

**Figure 2:**
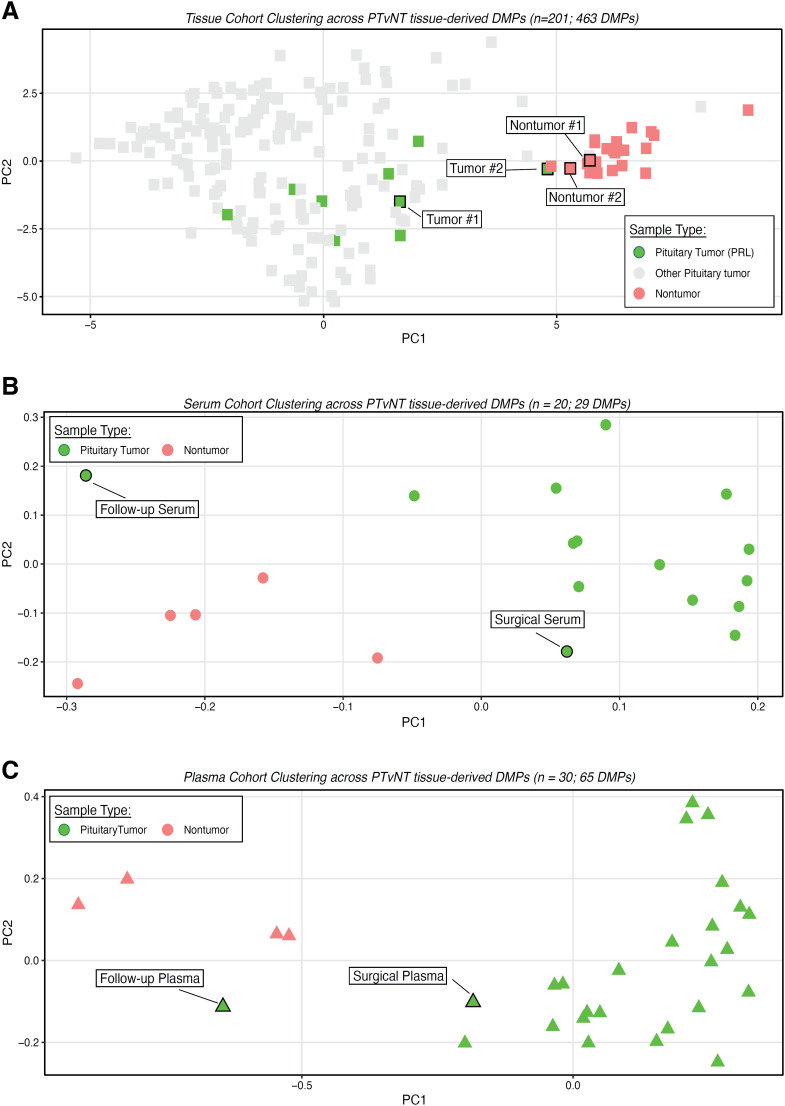
(**A**) Principal component analysis (PCA) displaying pituitary tumor-specific differentially methylated probes (DMPs) across a publicly available reference methylome cohort from pituitary tumor (PT) and nontumor (NT) controls and the patient's tumors (#1 and #2) and nontumor (#1 and #2) specimens. (**B-C**) PCA showing pituitary tumor-specific DMPs derived from the comparison between PT and NT methylome depicted in panel 2A, refined across the serum (**B**) and plasma (C) methylome from a publicly available cohort of PTs. Alignment of the patient's serum samples collected at surgery and during 12 months after surgery is highlighted.

We identified 463 PT-specific differentially methylated probes (DMPs or signatures) that significantly separated PTs and nontumor tissue-reference groups, as depicted in the PCA visualizations (Figure 2). These signatures clustered our patient's specimens according to their memberships, *i.e.*, Nontumor #1 and #2 specimens with the reference nontumor tissue; Tumor #1, containing 90% of tumor cells, with the reference tumor cohort and Tumor #2, containing a mixture of tumor and nontumor cells (20% and 80%, respectively) positioned closer to nontumor samples (Figure 2A). Among those signatures, 29 and 65 DMPs were captured across serum and plasma specimens (p ≤ 0.05 and ≤ 0.01), respectively, which similarly separated the patient's blood samples according to their memberships, *i.e.*, as tumor at diagnosis and nontumor at the 12-month follow-up, coinciding with the normalization of prolactinemia after successful resection of both tumors (Figures 2B and 2C).

While the majority of the literature concerning the detection of cfDNA in blood-based liquid biopsy assessments primarily focuses on using plasma samples, attributed to their perceived lower risk of contamination with genomic DNA and dilution of cfDNA, our case revealed that both serum and plasma exhibited similar capability in capturing PT-specific methylation signatures and distinguishing between tumor and nontumor samples—thereby corroborating our previous findings (16,17,22).

### Data availability

The raw DNA methylation level data files (EPIC Array; *.idat) for tumor tissue-reference molecular data analyzed in this study, compiled from Gene Expression Omnibus (GEO) under accession codes GSE109381, GSE90496, GSE54415, GSE115783 (23), and liquid biopsy specimen data, including 13 that matched pituitary tissue data generated at Henry Ford Health, have been deposited to Mendeley Data (https://data.mendeley.com/datasets/x954653zkr/1) (17,23).

## DISCUSSION

Multiple PTs are rare entities that mostly involve a combination of different pituitary adenoma subtypes, with GH- and ACTH-secreting tumors being the most common (25). Reports of multiple PTs composed of the same pituitary adenoma subtypes are infrequent and involve mainly the co-occurrence of gonadotroph tumors (3-10). In other reports, double lactotroph tumors were suspected based on hormonal and imaging assessment, and in one of them, they were diagnosed on the pathology report (4,10,25). Here, we described a case of double lactotroph tumors confirmed by postsurgical pathology and molecular assessments.

Multiple PTs commonly present as contiguous lesions, posing challenges in ir detection during preoperative MRI assessment, which is currently considered the gold standard for diagnosing isolated and multiple PTs. Even during surgical inspection, these tumors may go unnoticed and only become evident during postoperative pathological examinations (6,9,25). Failure to remove these tumors completely may lead to a misdiagnosis of treatment resistance, underscoring the importance of recognizing their presence.

Both intraoperative MRI (iMRI) and ultrasonography (IOUS) are valuable modalities for locating central nervous system (CNS) lesions during surgery, each with specific limitations and selected indications. While iMRI may not always be readily available and can extend anesthesia and operative time, it can also present technical challenges related to neuronavigational accuracy and susceptibility to brain shift during surgery (11,15). Conversely, IOUS allows for real-time adjustments to accommodate surgical tissue movement. However, its image quality is generally inferior to the high-resolution iMRI counterpart, and its accuracy relies on the operator's skill and experience in ultrasonography for proper interpretation. Studies have demonstrated that IOUS can lead to shorter operative times, reduced blood loss, shorter hospital stays, and a higher rate of gross total resection in patients undergoing transsphenoidal surgery for PTs (12,13,15). In the current case, preoperative MRI identified separated double PTs. However, during intraoperative visual inspection, the smallest tumor went undetected, which was overcome by the utilization of IOUS. This enabled the precise localization of the smaller lesion and facilitated the gross total resection of both tumors.

Recent studies, including our own and those conducted by others, have underscored the pivotal role of DNA methylation patterns detected in tissue specimens for the development of methylation classifiers (21,23). Due to the robustness of this approach, many centers, during tumor board decisions, have implemented and integrated a methylation-based CNS tumor classifier as a diagnostic tool, resulting in the resolution of challenging diagnostic cases of CNS tumors (20). In line with the tissue findings, we and others have shown that a methylation-based classifier is also feasible for liquid biopsy specimens and significantly differentiates across CNS tumors, including those that affect the sella turcica (16-18,22). For instance, we have demonstrated that blood-derived methylation markers effectively discriminated PTs from other sellar tumors (*e.g.*, craniopharyngioma) and other CNS tumors (*e.g.*, meningiomas) (16-18,22), suggesting the potential of liquid biopsy as a noninvasive diagnostic tool. Corroborating with our previous findings, in the present case, we successfully identified and differentiated PT and nontumor specimens through the utilization of PT-specific methylation signatures using tissue and liquid biopsy specimens (Figures 2A-C).

Of note, plasma is typically favored over serum for molecular profiling in liquid biopsy due to its lower risk of contamination with DNA from lysed white blood cells. However, serum samples, despite their higher risk of contamination, do not appear to interfere with the detection of tumor-specific methylation markers using sensitive methods like the EPIC array, as demonstrated in the present case and in our previous CNS studies (16-18,22). Nonetheless, a systematic comparison between methylation-based results obtained from serum and plasma in a large patient cohort is still warranted for confirmation of these results.

For most PTs, including those described herein, the integration between clinical and imaging features and hormonal markers detected in biofluids (blood, saliva, urine) is sufficient for an accurate presurgical diagnosis, which is usually confirmed through pathological assessment of tumor tissue (immunohistochemistry for adenohypophyseal hormones and/or cell lineage-specific transcription factors) (2). Nevertheless, certain rare sellar masses, such as primary lymphoma or metastasis, may mimic PTs in presurgical imaging. In these challenging cases, the use of complementary noninvasive methods like liquid biopsy could be beneficial in guiding a more specific treatment, such as chemotherapy and/or radiotherapy (28,29). Furthermore, as an illustrative example of the potential utility of methylation-based liquid biopsy in disease monitoring and treatment response assessment (16), we observed that during the 12-month follow-up, concurrent methylation profiling, and assessment of prolactin levels in the blood indicated that methylation markers clustered with nontumor specimens (Figure 2B-C), coinciding with normalization of prolactinemia levels, implying successful tumor control after surgery.

While liquid biopsy molecular markers may not offer additional benefits when reliable and cost-effective hormonal markers are available, they could be potentially valuable for detecting early recurrence in nonfunctioning PTs that lack specific biochemical markers, as they can distinguish these tumors from other sellar masses and potentially identify therapeutic markers. Despite their promise, these potential applications require confirmation in larger studies. Ongoing advancements in liquid biopsy-based molecular profiling, particularly leveraging methylation markers, are propelling the development of robust, cost-effective, and widely accessible diagnostic and monitoring tools for numerous tumors using a blood draw (30).

In conclusion, this study underscores the importance of IOUS in detecting tumors missed through intraoperative visual inspection, particularly in the context of multiple PTs. Additionally, it highlights the utilization of tumor-specific methylation markers, detected in the blood, aiding in the diagnosis and monitoring of dynamic changes in response to surgical treatment for PTs, as shown in other CNS tumors (16-18,22). Broadly, this study emphasizes the significance of intraoperative imaging and the translation of basic research (detection of methylation markers) in improving patient management and quality of care.
